# Screening for left ventricular hypertrophy in patients with type 2 diabetes mellitus in the community

**DOI:** 10.1186/1475-2840-10-29

**Published:** 2011-04-14

**Authors:** Jithendra B Somaratne, Gillian A Whalley, Katrina K Poppe, Mariska M ter Bals, Gina Wadams, Ann Pearl, Warwick Bagg, Rob N Doughty

**Affiliations:** 1 Cardiovascular Research Group, Department of Medicine, Faculty of Medical and Health Sciences, The University of Auckland, Private Bag 92019, Auckland Mail Centre, Auckland 1142, New Zealand

## Abstract

**Background:**

Left ventricular hypertrophy (LVH) is a strong predictor of cardiovascular disease and is common among patients with type 2 diabetes. However, no systematic screening for LVH is currently recommended for patients with type 2 diabetes. The purpose of this study was to determine whether NT-proBNP was superior to 12-lead electrocardiography (ECG) for detection of LVH in patients with type 2 diabetes.

**Methods:**

Prospective cross-sectional study comparing diagnostic accuracy of ECG and NT-proBNP for the detection of LVH among patients with type 2 diabetes. Inclusion criteria included having been diagnosed for > 5 years and/or on treatment for type 2 diabetes; patients with Stage 3/4 chronic kidney disease and known cardiovascular disease were excluded. ECG LVH was defined as either the Sokolow-Lyon or Cornell voltage criteria. NT-proBNP level was measured using the Roche Diagnostics Elecsys assay. Left ventricular mass was assessed from echocardiography. Receiver operating characteristic curve analysis was carried out and area under the curve (AUC) was calculated.

**Results:**

294 patients with type 2 diabetes were recruited, mean age 58 (SD 11) years, BP 134/81 ± 18/11 mmHg, HbA_1c _7.3 ± 1.5%. LVH was present in 164 patients (56%). In a logistic regression model age, gender, BMI and a history of hypertension were important determinants of LVH (p < 0.05). Only 5 patients with LVH were detected by either ECG voltage criteria. The AUC for NT-proBNP in detecting LVH was 0.68.

**Conclusions:**

LVH was highly prevalent in asymptomatic patients with type 2 diabetes. ECG was an inadequate test to identify LVH and while NT-proBNP was superior to ECG it remained unsuitable for detecting LVH. Thus, there remains a need for a screening tool to detect LVH in primary care patients with type 2 diabetes to enhance risk stratification and management.

## Background

The complications of type 2 diabetes are common and largely account for the excess morbidity and mortality associated with this disease. As such routine screening of asymptomatic patients with type 2 diabetes for retinopathy, nephropathy and neuropathy is recommended [1]. Diabetes is a major risk factor for coronary heart disease and cardiovascular disease is the most important cause of morbidity and mortality in patients with type 2 diabetes, accounting for approximately two-thirds of total mortality [2]. In addition, type 2 diabetes mellitus is associated with a cardiomyopathy characterised by left ventricular hypertrophy (LVH) and diastolic dysfunction [3]. However, current guidelines do not recommend routine screening for structural heart disease in these patients [1].

Diabetes is associated with LVH, left ventricular (LV) diastolic dysfunction [4,5], LV systolic dysfunction and cardiac autonomic neuropathy [3]. A large proportion of patients with type 2 diabetes and no known cardiovascular disease have LVH [6]. LVH is an important risk factor for cardiovascular disease in the general population [7]. Regression of LVH by pharmacological intervention is associated with an improvement in prognosis [8,9]. Therefore detection of LVH is attractive given the high prevalence and hence high pre-test probability of LVH in patients with type 2 diabetes.

In clinical practice the most reliable tool for quantifying left ventricular mass and diagnosing LVH is transthoracic echocardiography. Conversely the electrocardiogram (ECG), though inexpensive and widely available, is of limited use in detecting LVH in patients with type 2 diabetes due to its low sensitivity [10]. As both ECG and echocardiographic tools are currently unsuitable for wide population screening, there is a need for an accessible, acceptable, and economical test for detecting LVH in such patients. Identification of patients with LVH would facilitate the commencement of therapies that reduce LV mass and hence improve outcome. A biomarker, such as N-terminal pro brain natriuretic peptide (NT-proBNP), may be the solution. This simple and relatively inexpensive test can be measured from a non-fasting venous blood sample. NT-proBNP is released from the heart under conditions of increased wall stress [11] and is used primarily in the diagnosis of heart failure in patients with dyspnoea. Amongst patients with type 2 diabetes, higher BNP levels were observed in those with LVH [12].

Recent data suggests that NT-proBNP may be of value in identification of LVH among patients with hypertension presenting to emergency departments [13]. However, to date there have been no studies comparing diagnostic accuracy between NT-proBNP and ECG for detection of LVH in patients with type 2 diabetes. This study aimed to determine the value of NT-proBNP in the detection of LVH among patients with type 2 diabetes and no known cardiovascular disease in primary care.

## Methods

In this investigator-initiated study, all researchers were independent of the funding bodies and had complete access to all data. Ethics approval was obtained from the Northern Y Regional Ethics Committee (New Zealand).

During a 14-month period (March 2006 - May 2007), 294 patients with type 2 diabetes diagnosed by their general practitioner, of at least 5 years duration and/or on treatment for type 2 diabetes, were prospectively recruited from primary care. Patients with known cardiac disease (including coronary heart disease, heart failure, LVH (identified on previous ECG or echo done for clinical purposes), moderate and severe valvular heart disease, atrial fibrillation), cerebrovascular disease (prior stroke or transient ischaemic attack), peripheral arterial disease, Stage 3 chronic kidney disease (eGFR < 60 mL/min) or inability to provide informed consent were excluded. A general practitioner (GP) network previously developed in the Natriuretic Peptides in the Community study [14] was used to facilitate recruitment of primary care patients. Patients meeting study inclusion and exclusion criteria were identified and referred to the study centre by 51 participating GPs within the Auckland region. Study personnel contacted referred patients, provided further details regarding the study and invited them to a study visit.

All patients were seen and evaluated in the Cardiovascular Research Clinic at The University of Auckland. During the study visit patient eligibility was confirmed and informed consent was obtained. Basic demographics and medical history including information regarding known microvascular complications of type 2 diabetes were recorded. The mean of three seated blood pressure (BP) measurements, separated by a minimum interval of five minutes, was obtained. Height, body mass, waist circumference, hip circumference, and body composition were measured. Blood was collected using standard venepuncture technique and samples were sent to a tertiary referral medical laboratory for measurement of creatinine, glucose, HbA_1c_, lipids, and NT-proBNP. A single urinary albumin:creatinine ratio measured within 12 months of the study visit was obtained from community laboratories. If this was unavailable, participants were directed to have this done soon after the study visit.

All patients had a resting transthoracic echocardiogram (Philips HDI 5000/iE33, Bothell, Seattle, Washington) which was the reference standard for the detection of LVH in this study performed by a research-trained sonographer. LV mass was assessed from M-mode images in accordance with The American Society of Echocardiography (ASE) guidelines [15]. When M-mode images were unsuitable for measurement, 2-dimensional images were used. The LV mass gender-specific cut-offs for LVH used were: >45 g/m^2.7 ^in women and >49 g/m^2.7 ^in men[15]. All echocardiographic measurements were made by a cardiologist with subspecialty training in echocardiography. For LV mass the coefficient of variability for intra-observed repeated measures is less than 8% [16]. The echocardiographer and cardiologist measuring the images were blinded to ECG and NT-proBNP results.

All patients had a standard unfiltered 12-lead ECG (Philips Hewlett-Packard PageWriter 200 Cardiograph, Andover, Massachusetts). Each ECG was measured by a single analyst using a 150 mm digital vernier calliper under a five-fold magnification. ECG criteria used for the detection of LVH included the Sokolow-Lyon (SV_1 _+ RV_5/6_)[17] and Cornell (women RaVL + SV_3 _+ 0.8 mV; men RaVL + SV_3_)[18] voltage criteria. Standard cut-offs for the electrocardiographic diagnosis of LVH were used: Sokolow-Lyon voltage >3.5 mV[17] and Cornell voltage >2.8 mV[18]. Patients meeting either criterion were considered to have LVH by ECG. The ECG analyst was blinded to echocardiographic and NT-proBNP results. Thirty participants were randomly selected for estimation of test reproducibility. This analysis demonstrated an intra-observer and inter-observer variability of ≤0.01 mV for measurement of ECG voltages.

NT-proBNP levels were measured using the Roche Diagnostics Elecsys assay (pmol/L). The performance characteristics claimed by the manufacturer are an analytical sensitivity of 0.6 pmol/L and functional sensitivity of <5.9 pmol/L.

## Statistical methods

In the Casale Monferrato Study [19] the prevalence of ECG LVH was 17%. Assuming 90% power approximately 200 patients with diabetes and no LVH and 50 patients with diabetes with LVH would resolve an area under a Receiver Operating Characteristic (ROC) curve (AUC) of 0.8 and 0.9 between ECG LVH and NT-proBNP. To allow for information not being available from all patients the sample size was 300 subjects.

Normally distributed variables are presented as mean (standard deviation) and significantly skewed variables as median (interquartile range). Receiver operating characteristic (ROC) curve analyses were performed to evaluate the diagnostic performance of NT-proBNP in the detection of LVH, with sub-analyses on the basis of age, gender, BMI and albuminuria. The area under the curve (AUC) was used to assess the discriminative ability of NT-proBNP. Sensitivity, specificity, predictive values and the positive likelihood ratio are calculated at the threshold determined by the maximal Youden index.

Multivariable logistic regression was used to investigate the relationship between patient factors and LVH. Variables were selected for inclusion in the model on the basis of biological plausibility. The natural logarithm of NT-proBNP was used to satisfy model assumptions.

Analyses were performed using SAS 9.1 (SAS Institute Inc, Cary, NC, USA) statistical software.

## Results

**Figure 1:**
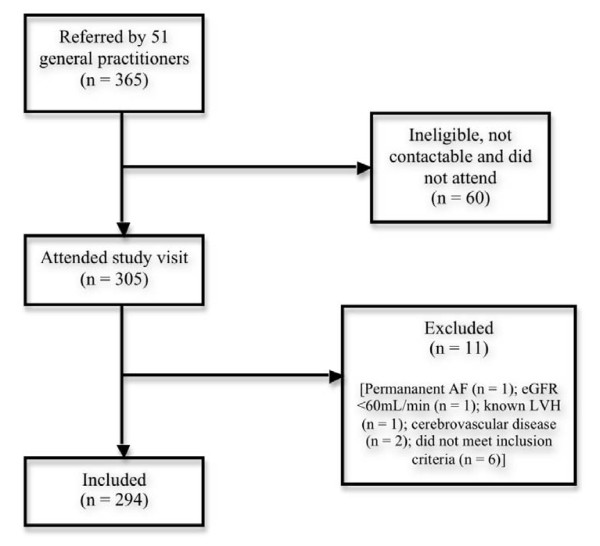
**Participant flow**. Abbreviations: AF atrial fibrillation, eGFR estimated glomerular filtration rate, LVH left ventricular hypertrophy

Of the 365 potential participants referred by 51 general practitioners, 60 were ineligible, uncontactable or did not attend their study visit (Figure 1). A further 11 participants were excluded due to permanent atrial fibrillation, Stage 3 chronic kidney disease, known LVH, previous stroke or failing to satisfy inclusion criteria. The remaining 294 participants were included in the study. The mean age of participants was 58 ± 11 years, 49% were women, 42% were Caucasian, 33% were Polynesian (Mäori and Pacific Islander) and 25% were Asian. Mean body mass index (BMI) was 31.9 ± 7.0 kg/m^2 ^and mean BP 134/81 ± 18/11 mmHg. Median time since diagnosis of type 2 diabetes was 6 years (range 1 month - 50 years). Mean HbA_1c _7.3 ± SD 1.5% and median urinary albumin:creatinine ratio (UACR) was 1.2 (IQR 0.4, 4.0) mg/mmol. One hundred and eighty nine (66%) participants had a normal UACR (<2.5 mg/mmol), 72 (25%) had microalbuminuria (UACR 2.5 - 20 mg/mmol) and 27 (9%) had macroalbuminuria (UACR >20 mg/mmol). A history of known retinopathy was noted in 14%, nephropathy in 11% and neuropathy in 8%. Many participants had a history of other cardiovascular risk factors such as hypertension (60%) and dyslipidaemia (70%). Half of all participants were prescribed aspirin, 61% a statin and 47% an angiotensin converting enzyme (ACE) inhibitor. Nearly all patients were on pharmacological therapy for type 2 diabetes (94%): 244 were on oral hypoglycaemic therapy alone, 6 on subcutaneous insulin therapy alone and 25 on both. Of the 269 participants on oral hypoglycaemic therapy, 243 (90%) were on metformin, 130 (48%) on a sulphonylurea, 8 (3%) on a thiazolidinedione and 1 on acarbose. (See Table 1)

**Table 1:** Patient characteristics

***Clinical***	
Female, n	145 (49%)
Age, years (SD)	58 (11)
Median duration of diabetes, months (IQR)	72 (36, 120)
History of hypertension, n	175 (60%)
History of dyslipidaemia, n	206 (70%)
Body mass index, kg/m^2 ^(SD)	31.9 (7.0)
Systolic blood pressure, mmHg (SD)	134 (18)
Diastolic blood pressure, mmHg (SD)	81 (11)
HbA_1c_, % (SD)	7.3 (1.5)
Median UACR, mg/mmol (IQR)	1.2 (0.4, 4.0)
Normal UACR (<2.5 mg/mmol), n	189 (66%)
Microalbuminuria (UACR 2.5 - 20 mg/mmol), n	72 (25%)
Macroalbuminuria (UACR ≥20 mg/mmol), n	27 (9%)
***Current therapy***	
Oral hypoglycaemic therapy, n	269 (91%)
Subcutaneous insulin therapy, n	31 (11%)
Any antihypertensive therapy, n	190 (65%)
Statin therapy, n	178 (61%)
***Echocardiographic characteristics***	
Left ventricular mass, g (SD)	207 (63)
Left ventricular mass index, g/m^2.7 ^(SD)	51.5 (14.6)
Left ventricular hypertrophy, n	164 (56%)
Mild, n	52 (18%)
Moderate, n	49 (17%)
Severe, n	63 (21%)
Regional wall motion abnormality, n	17 (6%)
Left ventricular systolic dysfunction, n	12 (4%)
Left ventricular diastolic dysfunction, n	260 (89%)
Abnormal relaxation, n	173 (59%)
Pseudonormal filling, n	87 (30%)

Values represent mean unless stated. **Abbreviations**: *IQR *interquartile range, *SD *standard deviation, *UACR *urinary albumin:creatinine ratio

The mean LV mass index was 51.5 ± 14.6 g/m^2.7^. LVH was diagnosed by echocardiography in 164 of the 294 participants (56%). Using the ASE partition values of LV mass index [15], 52 participants (18%) had mild LVH (women 45-51 g/m^2.7^, men 49-55 g/m^2.7^), 49 (17%) moderate LVH (women 52-58 g/m^2.7^, men 56-63 g/m^2.7^) and 63 (21%) severe LVH (women ≥59 g/m^2.7^, men ≥64 g/m^2.7^). Of the 119 participants with no previous history of hypertension, 44 (37%) had LVH. Participants on either an ACE inhibitor or angiotensin receptor blocker were more likely to have LVH (62% vs. 49%). The European Society of Hypertension (ESH) and European Society of Cardiology (ESC) 2007 Guidelines for the Management of Arterial Hypertension use LV mass index thresholds of 110 g/m^2 ^for women and 125 g/m^2 ^for men to diagnose LVH [20]. By this more conservative definition, 79 (27%) had LVH. (See Table 1)

Important incidental findings on echocardiography included a resting regional wall motion abnormality in 17 (6%) and LV systolic dysfunction in 12 (4%). LV diastolic dysfunction, as assessed by mitral filling pattern, demonstrated abnormal relaxation in 173 (59%) and pseudonormal filling in 87 (30%) of patients. (See Table 1)

### Detection of left ventricular hypertrophy

Only 4 of the 164 participants (2%) with known echocardiographic LVH were correctly detected by either the Sokolow-Lyon or Cornell voltage criteria. The sensitivity of ECG for detecting echocardiographic LVH was 2% (95% CI 1 to 6%) with a specificity of 99% (95% CI 96 to 100%).

**Figure 2:**
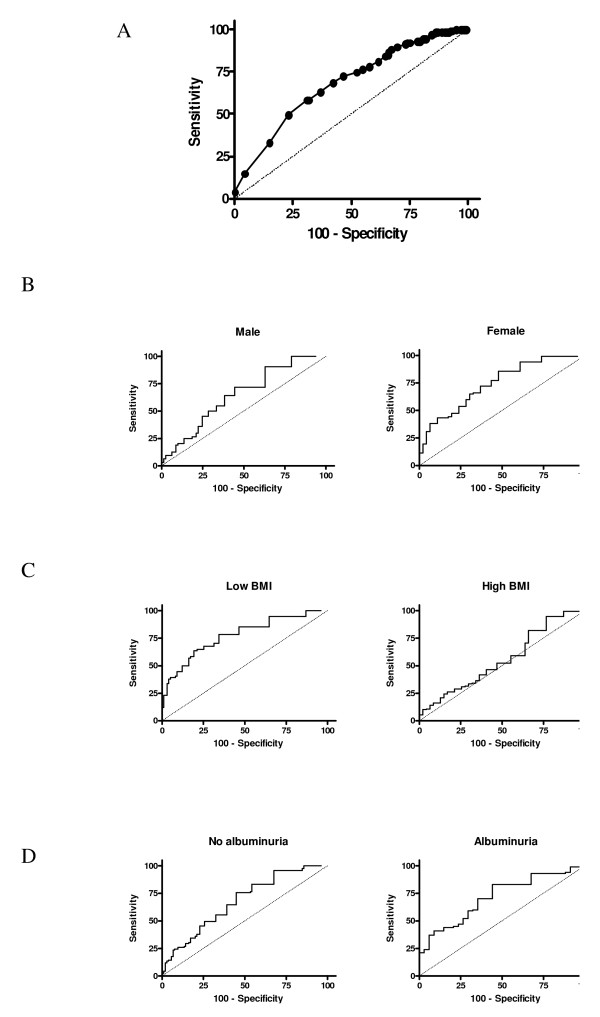
Receiver operating characteristic curves for NT-proBNP in discriminating left ventricular hypertrophy for whole group (A) and according to: gender (B); body mass index (BMI, C); presence of albuminuria (urinary albumin:creatinine ratio ≥2.5 mg/mmol, D)

The median NT-proBNP level was 6.0 pmol/L (range of <0.6-175.0). The area under the curve (AUC) for NT-proBNP in discriminating patients with LVH from those with no LVH was 0.68 (95% CI 0.62 to 0.74) (Figure 2). The maximum Youden index was established at an NT-proBNP level of 4.4 pmol/L (37.3 pg/mL). At this cut off the sensitivity was 68% (95% CI 60 to 75%) and the specificity was 58% (95% CI 50 to 68%). There was no improvement in the performance of NT-proBNP in distinguishing between patients with moderate-severe LVH and those with no LVH-mild LVH (AUC 0.64). Similarly, using the ESH/ESC definition of LVH, there was no difference in the utility of NT-proBNP in identifying patients with LVH (AUC 0.64). When participants were categorised into three groups [normal UACR (<2.5 mg/mmol), microalbuminuria (UACR 2.5 - 20.0 mg/mmol) and macroalbuminuria (UACR >20 mg/mmol)] the median NT-proBNP level was similar: 6.0 (IQR 3.0 - 12.0) pmol/L, 6.0 (IQR 3.0-16.5) pmol/L and 8.0 (IQR 3.0 - 21.0) pmol/L respectively.

There was some variability in the diagnostic accuracy of NT-proBNP between subgroups of participants (Figure 2). For example, the AUC was higher in women (0.72 vs. 0.61) and in those with a BMI below the median of 30.8 kg/m^2 ^(0.78 vs. 0.56). There was no difference in AUC for patients with UACR ≥2.5 mg/mmol (0.68) and <2.5 mg/mmol (0.69).

In a multivariable model including age, gender, history of hypertension, time since diagnosis of type 2 diabetes, BMI, HbA_1c_, the presence of albuminuria, and log_e _(NT-proBNP), the significant determinants of LVH were: BMI (χ^2 ^24.7; <0.001), age (χ^2 ^8.3; p = 0.004), history of hypertension (χ^2 ^4.9; p = 0.028), and gender (χ^2 ^4.1; p = 0.043).

## Discussion

This study, involving a group of patients with type 2 diabetes with no known cardiac, cerebrovascular or peripheral vascular disease, has demonstrated that LVH (defined according to the ASE guidelines [15]) was common, occurring among 56% of the patients. The detection of LVH by standard ECG criteria was poor and while NT-proBNP was superior to ECG in discriminating LVH, it remains unsuitable for use as a screening tool due to inadequate optimum sensitivity and specificity.

### Clinical importance of left ventricular hypertrophy

The prevalence of LVH in patients with type 2 diabetes has varied considerably based on the mode of detection and baseline characteristics of the cohort studied. The high proportion (56%) of participants with LVH in this study is comparable with previously reported data. For example, echocardiographic LVH was found in 22% [12] and 51% [21] of Australian diabetes clinic attendees. A higher prevalence of echocardiographic LVH (71%) was noted in diabetes clinic attendees in Dundee, Scotland [22], although a lower proportion (9.4%) among patients with diabetes without known macrovascular complications in a recent study from Sweden [4]. In our study 37% of participants with no history of hypertension had LVH; suggesting that type 2 diabetes per se is associated with LVH. More importantly the clinically prevalent combination of type 2 diabetes and hypertension was strongly linked to the development of LVH, occurring in 69% of such patients.

The relationship between LVH and poor prognosis [7] would suggest that this common finding in patients with type 2 diabetes is clinically significant. For example in the Reduction of End Points in Non-insulin Dependent Diabetes Mellitus with the Angiotensin II Antagonist Losartan (RENAAL trial), LVH was associated with an increased risk of death, end-stage renal disease and doubling of serum creatinine (hazard ratio 1.41; p < 0.001) in patients with type 2 diabetes, clinical nephropathy and no known cardiovascular disease [23].

LVH is emerging as an important independent therapeutic target. Treatment with losartan was associated with a reduction in ECG LVH in the RENAAL trial [23]. A change in LVH voltage criteria was an independent predictor of cardiovascular events in hypertensive patients with diabetes in the Appropriate Blood Pressure Control in Diabetes (ABCD) trial [24]. These data provide some hope that, in asymptomatic patients with type 2 diabetes, the aggressive treatment of LVH per se or risk factors for LVH, such as hypertension, may yield significant long term prognostic benefits. A substudy of the Losartan Intervention For Endpoint Reduction in Hypertension (LIFE) study focussing on patients with diabetes suggested that patients with diabetes experienced less regression of LVH in response to losartan and any regression was not predictive of future cardiovascular events [25]. The increased formation of advanced glycation end products and their cross-linking with myocardial collagen associated with diabetes was proposed as a mechanism to account for this difference. This highlights the need to develop other therapies specifically targeting LVH, independent of BP lowering, such as advanced glycation end product-protein breakers [26].

### Detection of left ventricular hypertrophy

#### ECG

The 12-lead ECG, the most commonly used tool for diagnosis of LVH in the community, performed poorly in detecting LVH compared to echocardiography. The most widely used ECG criteria are the Sokolow-Lyon voltage criteria. Using these criteria, the prevalence of LVH in this cohort would only be 1%. The combination of both the Sokolow-Lyon and the Cornell voltage criteria only raised the prevalence to 1.7%. Similarly in the Scottish study of diabetes clinic patients, in whom the prevalence of echocardiographic LVH was 71%, the prevalence of ECG LVH using the LIFE criteria was only 9.2% [22]. The poor performance of ECG in detecting LVH, using voltage-based criteria, may result from the attenuation of electrocardiographic voltages at the skin surface by increased fat mass in obese individuals [27]. In our study participants with a higher BMI were found to have a higher prevalence of echocardiographic LVH (73% vs. 39%), yet lower mean Sokolow-Lyon voltages (1.7 mV vs. 1.9 mV) and the same mean Cornell voltages (1.2 mV).

#### NT-proBNP

Though NT-proBNP was far superior to ECG in detecting LVH it was inadequate for general use as a screening tool for LVH in community patients with type 2 diabetes and no overt cardiovascular disease. One possible explanation for the poor performance of NT-proBNP in detecting LVH in this study relates to the low levels of NT-proBNP levels (median 6.0 pmol/L) in comparison to diagnostic cut-offs for heart failure [14]. This may relate to the both the obese nature of this cohort (mean BMI 31.9 kg/m2) as well as the prevalence of metabolic risk factors. Metabolic risk factors have previously been independently associated with lower natriuretic peptide levels [28]. Thus the release of NT-proBNP promoted by LVH in our patients may well have been dampened by the counterinfluence of obesity and metabolic risk factors. Recently data has suggested that a combined approach using ECG and NT-proBNP can improve identification of LVH among patients with hypertension presenting to emergency departments [13]. However, this study was from a small number of patients (49) among whom 43% had LVH from the ECG and while promising these results would need to be confirmed in a larger study and this approach has not been evaluated among patients with diabetes.

The functional sensitivity, the lowest concentration that can be reliably measured with a between-run coefficient variation of 20%, of the NT-proBNP assay (<5.9 pmol/L) employed in this study may have significantly decreased the performance of NT-proBNP in detecting LVH. Approximately half of this cohort had a NT-proBNP measurement below the functional sensitivity. The imprecise nature of the assay at these low concentrations may conceal the true relationship between LVH and NT-proBNP level in this cohort. The optimal statistical NT-proBNP cut-off for the detection of LVH (4.4 pmol/L using the maximum Youden index) is not clinically useful as it well below the functional sensitivity of the available assay.

### Future approaches

Given the poor performance of current ECG LVH criteria and NT-proBNP in detecting echocardiographic LVH there is a need for the development of alternative methods for detecting this prevalent complication which is associated with an adverse prognosis. The main limitation of current voltage-based ECG LVH criteria appears to be the attenuation of electrocardiographic voltages at the skin surface by subcutaneous fat. Indexing voltages to measures of body composition, such as body fat percentage, may help adjust for the attenuation of voltages by increased body fat and increase the utility of current voltage criteria in obese individuals. At present the Framingham-adjusted Cornell voltage criteria is the only available ECG LVH criteria adjusting for a measure of body size by incorporating BMI [29]. Other voltage-independent ECG LVH, such as QRS duration and QT interval, require further investigation this population.

Another possible tool for the detection of LVH in the community is hand-carried echocardiography (HCE). This is a cheaper, user-friendly and more accessible alternative to standard transthoracic echocardiography. A limited echocardiographic study using HCE may be a valuable screening tool for not just LVH but also resting regional wall motion abnormalities, significant LV systolic and diastolic dysfunction in a group of asymptomatic individuals at high risk of cardiovascular disease. This would require further prospective evaluation.

### Limitations

Systematic assessment for silent myocardial ischaemia was not assessed in this study and hence its confounding influence is uncertain. Chronic stable coronary heart disease is known to be associated with higher natriuretic peptide levels[30]. Though patients with known coronary heart disease were excluded from our study we did not assess or exclude patients with silent myocardial ischaemia. While silent myocardial ischaemia may have been a confounding factor we believe that it has not significantly altered the overall results or conclusions of this study given the low NT-proBNP levels observed.

The quality of ECG recordings is important for accurate detection of LVH. In this study all ECG recordings were performed by staff that were trained and supervised by a registered technologist and using a state-of-the-art machine. Furthermore, all measurements were performed by a single observer according to protocol and quality reviewed by a cardiologist. Lastly, it is possible that the failure to detect LVH was related to the over-estimation of LVH by echocardiography. This is unlikely since the echoes were performed according to a strict protocol and reviewed and measured by a trained echocardiologist, and the prevalence of LVH is similar to other published cohorts.

## Conclusion

In conclusion, LVH was highly prevalent in asymptomatic patients with type 2 diabetes. ECG was an inadequate test to identify LVH in these patients. NT-proBNP though superior to ECG remains unsuitable as a screening tool to detect LVH in patients with type 2 diabetes. There remains a need for a screening tool to detect LVH in patients with type 2 diabetes in primary care to enhance risk stratification and management.

## Competing interests

The authors declare that they have no competing interests.

## Authors' contributions

RND and GAW conceived of the study. JBS, AP, RND, MMterB, WB and GW were responsible for study design and acquisition of data. GAW and RND performed and analysed the echocardiograms. KKP undertook the statistical analysis. JBS drafted first version of the manuscript. All authors have been involved in drafting and revision of the manuscript for important intellectual content and have read and approved the final manuscript for publication.
